# Physiologically Based Pharmacokinetic (PBPK) Modeling to Predict PET Image Quality of Three Generations EGFR TKI in Advanced-Stage NSCLC Patients

**DOI:** 10.3390/ph15070796

**Published:** 2022-06-27

**Authors:** I. H. Bartelink, E. A. van de Stadt, A. F. Leeuwerik, V. L. J. L. Thijssen, J. R. I. Hupsel, J. F. van den Nieuwendijk, I. Bahce, M. Yaqub, N. H. Hendrikse

**Affiliations:** 1 Department of Clinical Pharmacology and Pharmacy, Amsterdam UMC Location Vrije Universiteit Amsterdam, Boelelaan 1117, 1081 HV Amsterdam, The Netherlands; a.f.leeuwerik@gmail.com (A.F.L.); j.r.i.hupsel@amsterdamumc.nl (J.R.I.H.); j.f.vandennieuwendijk@amsterdamumc.nl (J.F.v.d.N.); 2 Department of Pulmonary Medicine, Amsterdam UMC Location Vrije Universiteit Amsterdam, Boelelaan 1117, 1081 HV Amsterdam, The Netherlands; e.vandestadt@amsterdamumc.nl (E.A.v.d.S.); i.bahce@amsterdamumc.nl (I.B.); 3 Department of Radiation Oncology, Amsterdam UMC Location Amsterdam Medical Center, Meibergdreef 9, 1105 AZ Amsterdam, The Netherlands; v.thijssen@amsterdamumc.nl; 4 Department of Radiology and Nuclear Medicine, Amsterdam UMC Location Vrije Universiteit Amsterdam, Boelelaan 1117, 1081 HV Amsterdam, The Netherlands; maqsood.yaqub@amsterdamumc.nl (M.Y.); nh.hendrikse@amsterdamumc.nl (N.H.H.)

**Keywords:** NSCLC, EGFR TKI, PBPK modeling, PET/CT

## Abstract

Introduction: Epidermal growth factor receptor (EGFR) mutated NSCLC is best treated using an EGFR tyrosine kinase inhibitor (TKI). The presence and accessibility of EGFR overexpression and mutation in NSCLC can be determined using radiolabeled EGFR TKI PET/CT. However, recent research has shown a significant difference between image qualities (i.e., tumor-to-lung contrast) in three generation EGFR TKIs: ^11^C-erlotinib, ^18^F-afatinib and ^11^C-osimertinib. In this research we aim to develop a physiological pharmacokinetic (PBPK)-model to predict tumor-to-lung contrast and as a secondary outcome the uptake of healthy tissue of the three tracers. Methods: Relevant physicochemical and drug specific properties (e.g., pKa, lipophilicity, target binding) for each TKI were collected and applied in established base PBPK models. Key hallmarks of NSCLC include: immune tumor deprivation, unaltered tumor perfusion and an acidic tumor environment. Model accuracy was demonstrated by calculating the prediction error (PE) between predicted tissue-to-blood ratios (TBR) and measured PET-image-derived TBR. Sensitivity analysis was performed by excluding each key component and comparing the PE with the final mechanistical PBPK model predictions. Results: The developed PBPK models were able to predict tumor-to-lung contrast for all EGFR-TKIs within threefold of observed PET image ratios (PE tumor-to-lung ratio of −90%, +44% and −6.3% for erlotinib, afatinib and osimertinib, respectively). Furthermore, the models depicted agreeable whole-body distribution, showing high tissue distribution for osimertinib and afatinib and low tissue distribution at high blood concentrations for erlotinib (mean PE, of −10.5%, range −158%–+190%, for all tissues). Conclusion: The developed PBPK models adequately predicted the image quality of afatinib and osimertinib and erlotinib. Some deviations in predicted whole-body TBR lead to new hypotheses, such as increased affinity for mutated EGFR and active influx transport (erlotinib into excreting tissues) or active efflux (afatinib from brain), which is currently unaccounted for. In the future, PBPK models may be used to predict the image quality of new tracers.

## 1. Introduction

Lung cancer is one of the most prevalent cancer types with over 2 million new cases each year worldwide [1,2,3]. Chemotherapy has long been the sole treatment of metastatic nonsmall cell lung cancer (NSCLC), but over the past decade, targeted therapies against specific oncogenic driver pathways have been developed [4]. One of these oncogenic driver pathways is the epidermal growth factor receptor (EGFR) pathway [5]. An activating mutation in the kinase domain of the EGF receptor can lead to activation of the receptor. EGFR tyrosine kinase inhibitors (TKIs) block this activating pathway [5,6]. In recent years, three generations of EGFR-TKIs have been developed. First-generation EGFR inhibitors such as erlotinib bind reversibly to EGFR harboring mutations and to a lesser extent to wild-type EGFR [5,6,7]. To overcome resistance, second-generation TKIs such as afatinib were developed, which bind irreversibly to EGFR [6]. To achieve more selective binding to T790M mutant EGFR, third-generation TKIs such as osimertinib were developed [8]. These three generations of TKIs vary in potency and binding characteristics [9,10,11,12,13]. Treatment using EGFR-TKIs has shown better response rates and longer durations of response and has become the standard of care in patients where an activating EGFR mutation is present [1,3,14].

In recent years, research has been conducted using positron emission tomography (PET) to assess EGFR mutational status and to explore PET drug uptake as a predictive biomarker for response to EGFR-TKI treatment. Three generations of EGFR-directed PET tracers were developed by our group and the tracer uptake was studied in patients exposed to ^11^C-erlotinib, ^18^F-afatinib and ^11^C-osimertinib [15,16,17,18,19,20]. Such PET tracers can be used to predict whole-body and tumor drug uptake and thereby guide EGFR-TKI drug treatment. In a recent study, we compared published data from NSCLC patient scans of the three generations radiolabeled EGFR TKIs [21]. Three tracers were analyzed: ^11^C-erlotinib, ^18^F-afatinib and ^11^C-osimertinib. Tracer uptake was quantified using tumor-to-blood ratio (TBR). Previous research has shown that TBR is an adequate measure for quantification of tracer uptake in ^11^C-erlotinib and ^18^F-afatinib, and could also be applied for ^11^C-osimertinib [15,20,21]. Furthermore, tumor-to-lung contrast was used to assess PET image quality of each tracer. ^11^C-osimertinib showed a negative contrast: tumor-tissue uptake was 20% lower than surrounding lung tissue. In contrast, ^18^F-afatinib showed a better contrast, 96% higher in tumor tissue than in surrounding tissue. ^11^C-erlotinib image quality was deemed superior, with a tumor-to-lung contrast value of 178% [21].

**Figure 1:**
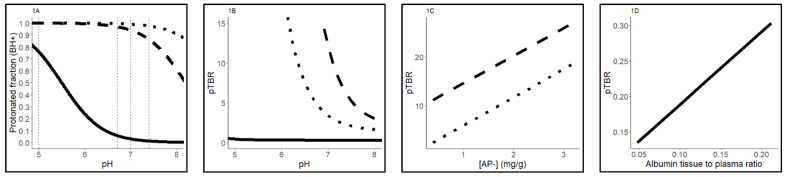

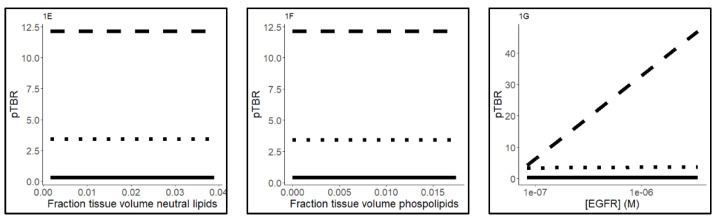
Schematic representation of the change in drug distribution in relation to relevant physiological values in human tissues. Uninterrupted lines represent erlotinib, dotted lines represent osimertinib and dashed lines represent afatinib. (**A**) pH versus protonation (Equation (A)). (**B**) pH of the intracellular water versus predicted TBR, tumor-to-blood ratio (Equation (B)). (**C**) AP-, acidic phospholipids versus predicted TBR (Equation (C)). (**D**) Albumin tissue-to-plasma ratio versus predicted TBR (Equation (D)). (**E**) and (**F**) fraction of neutral lipids and phospholipids versus predicted TBR (Equation (E)). (**G**) EGFR tissue concentration versus predicted TBR. pTBR: predicted tumor to blood ratio, BH: protonated base, AP-: acidic phospholipids, EGFR: epidermal growth factor receptor, Fiw: fraction intracellular water, B:P: blood to plasma partition coefficient of compound, Fu: fraction unbound drug of compound, pHiw: pH of intracellular water, pHp: pH of plasma, Fnp: fraction of neutral phospholipids, Fnl: fraction of neutral lipids, pKa: basicity, Ka: association constant.

Based on our analyses [21], we hypothesized that the physicochemical drug properties may explain some of the variability in penetration of the tracers in different tissues. Important physicochemical properties are lipophilicity (log P) and basicity, defined by the pKa (negative log of the acid dissociation constant or Ka value). Lipophilicity affects protein binding (e.g., albumin (ALB)) and transport and binding to neutral lipids and phospholipids (NL/NP) in the cell membranes. A strong basic drug (a compound with a high pKa), is highly protonated at physiological (plasma) pH levels, whereas weak bases (pKa < 7) are mostly unprotonated at physiological pH levels (Figure 1A). Protonation, whether an H^+^ atom is added to a base, affects transport over negatively charged acidic phospholipid membranes (Figure 1C). Unprotonated lipophilic drugs will bind to albumin (Figure 1D) and membrane NL/PL (Figure 1E,F).

Equations:

(1) $$Fraction\;BH^{+}=\frac{1}{1+10^{pKa-pH}}$$

(2) $$pTBR_{\mathrm{pHiw}}\;or\;contribution\;pH=\left(\frac{1+10^{pKa-pHiw}}{1+10^{pKa-pHp}}*fiw\right)*\frac{Fu}{B:P}$$

(3) $$pTBR_{\mathrm{AP-}}\;or\;AP-binding=\left(\frac{Ka*\left[AP-\right]*10^{pKa-pHiw}}{1+10^{pKa-pHp}}\right)*\frac{Fu}{B:P}\;$$

(4) $$pTBR_{\mathrm{ALB}}\;or\;Albumin\;binding=\left(Ka,ALB\;*\;\frac{\left[ALB,tissue\right]}{\left[ALB,plasma\right]}\right)*\frac{Fu}{B:P}$$

(5) $$pTBR_{\mathrm{Lipids}}\;or\;Lipid\;binding=\left(\frac{P*Fnl+\left(0.3P+0.7\right)*Fnp}{1+10^{pKa-pHp}}\right)*\frac{Fu}{B:P}\;$$

(6) $$pTBR_{\mathrm{EGFR}}\;or\;EGFR\;binding=\left(\frac{\frac{\left[EGFR\right]}{Kd}*\left(1+10^{pKa-pHiw}\right)}{1+10^{pKa-pHp}}*fiw\right)*\frac{Fu}{B:P}$$

Because of the differences in basicity, we hypothesize that lysosomal sequestration, or the trapping of the tracer in the lysosome, plays a role in the differences in tracer uptake. Lysosomes are acidic membrane-bound organelles which are capable of digesting biomolecules [22]. Since the pH level of the lysosome is lower (i.e., the lysosome is more acidic) than the cytosol, protonation will occur here to a greater extent than in the neutral environment of the cytosol (Figure 1A). When protonated transport through the lysosomal membrane decreases, it leads to “trapping” of the protonated base in the lysosome. Lysosomal sequestration is a well-known cellular distribution pattern and may cause intrinsic resistance to strong basic EGFR-TKIs due to decreased cytosolic concentrations and thereby less availability for EGFR binding [23,24].

In addition to EGFR abundance, other factors in the microenvironment of the tumor may predict the tumor-to-lung contrast. These compound- and tissue-specific properties may influence the whole-body distribution resulting in different tumor-to-lung contrast of the three compounds.

Biodistribution of small molecules, such as EGFR-TKIs, related to compound- and tissue-specific properties can be described using physiologically based pharmacokinetic models (PBPK). A physiologically based pharmacokinetic model (PBPK model) is a mathematical, mechanistical description of a physiological system where compartments are used to represent various tissues in the NSCLC population. Each compartment corresponds with physiological volumes of each tissue/organ [25]. Although PBPK models are frequently used in pharmacology research, no prior research has been done using PBPK models in relationship to PET tracers. An advantage of such mechanistic models is its predictive potential. In this study, we developed a PBPK model reflecting the essential features of tissue distribution of EGFR-TKIs with the primary aim to predict the image quality by predicting the right tumor-to-lung contrast. As a secondary aim, the model was used to predict the whole-body distribution. When fully validated, mechanistic PBPK models can be applied to predict tumor drug uptake in a wide array of diseases and structurally diverse compounds, and can be used to predict the image quality of new tracers—drugs with large tumor/tissue contrast.

## 2. Results

### 2.1. Components of the Mechanistical PBPK Model

The mechanistical PBPK model consists of the base model for each TKI, lysosomal sequestration for the strong bases (afatinib and osimertinib), tumor immune deprivation, unaltered tumor perfusion, EGFR target binding and a more acidic extracellular water (i.e., water located just outside the tumor cells in the tumor microenvironment) in tumor tissue. Each component added to the literature based on physicochemical base models was analyzed during the sensitivity analyses (below). The model, including unaltered perfusion but without a tumor vascularization coefficient, optimally predicted the tumor-to-lung contrast of all three EGFRs. See Supplemental Materials S-IV for full analysis of the mechanistical model including vascularization versus the final model excluding this component.

### 2.2. PBPK Model Validation Using PET Data

**Figure 2:**
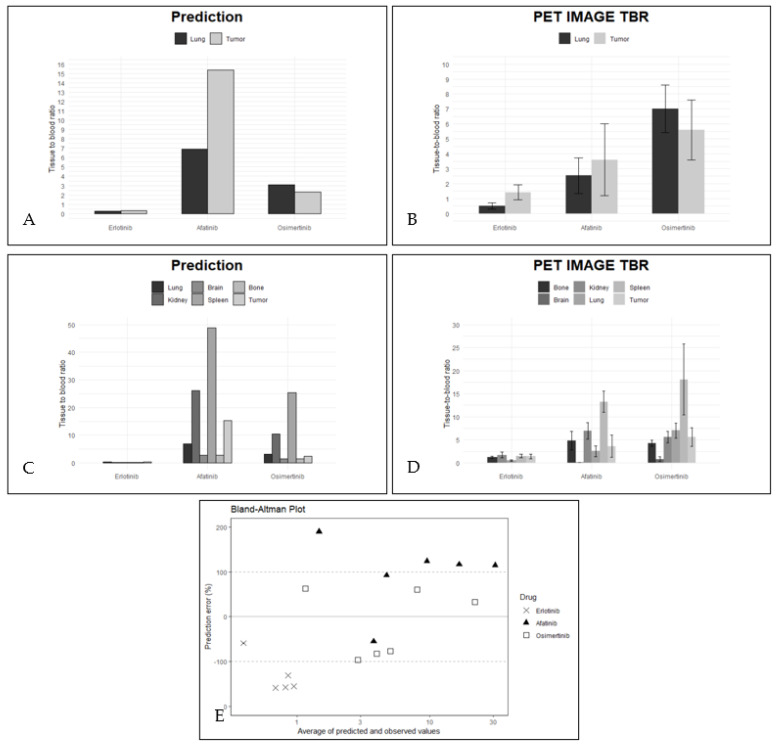
The association between PET-image-derived TBR and model-predicted TBR. (**A**) PET-image-derived TBR (left) vs. (**B**) model-predicted TBR of lung and tumor. (**C**) PET-image-derived TBR (left) vs. (**D**) model-predicted TBR of all tissues of interest. For patient data, standard deviations are given. (**E**) Bland–Altman plot showing accuracy of the model to predict tissue uptake. The solid black line represents the mean and the dashed lines a factor 3 of both sides of zero. Percentage of predictions falling within 3-fold: erlotinib 16.6%, afatinib 33.3% and osimertinib 100%.

The tumor-to-lung contrast was predicted using the mechanistic PBPK model to visualize differences in image quality observed in PET imaging, as shown in Figure 2A. The predicted TBR by using the final PBPK model are also shown in Figure 2B. Both observed and predicted TBR values showed high uptake of osimertinib and afatinib in lung and tumor tissue (TBR > 1) and high blood concentrations compared to tissue concentrations for erlotinib (TBR < 1, Figure 2A,B). The tumor-to-lung contrast for each EGFR TKI was predicted within the established boundaries, i.e., within threefold of the observed value. Furthermore, the model correctly predicts a tumor-to-lung contrast of >1 for erlotinib and afatinib, and <1 for osimertinib (Table 1).

Table 1 describes the contribution of different components in the mechanistic PBPK model. Erlotinib binds extensively to albumin in tissue, whereas osimertinib and afatinib predominantly bind to AP- in the cellular membranes and are sequestered in the lysosome. Substantial decrease in lysosomal sequestration was predicted for both strong basic EGFR-TKIs in the less lysosome-rich tumor in comparison with lung tissue. Tumor EGFR binding of afatinib was predicted to be extensive for and comprised 72.17% of all tissue binding, whereas for erlotinib and osimertinib the model showed that only a minor fraction of the tissue fraction bound to EGFR (1.89 and 1.85%, respectively). The whole-body distribution of the three EGFR-TKIs was described by the mechanistic PBPK models. The predicted TBR values by the mechanistic PBPK models are shown in Figure 2D compared to the measured TBR (obtained during PET imaging, Figure 2C).

As secondary outcome, the mechanistical PBPK model is able to predict the overall body distribution of the TKIs, with extensive distribution to most tissues for osimertinib and afatinib and limited tissue penetration for erlotinib. The observed and predicted TBR values of osimertinib and afatinib in most tissues were >1 and for erlotinib <1 (Figure 2C,D). The TBR predicted by the mechanistic PBPK model correlated strongly with the PET imaging data (r2: 0.593 with *p* < 0.0003; α = 0.01) with a mean PE of −10.5% (CI95% of the data: −126.0 to 104.9, Figure 2E). For afatinib, 33.3% of tissues were predicted within a factor of 3 of the observed value. The data point falling outside this range represented brain uptake of afatinib, and was predicted to be 189.7% times higher than observed, and tumor predictions above the 3-fold limit of the observed mean value (Table 2). However, for osimertinib, 100% of tissues were predicted to be within 3-fold of the observed tissue uptake of PET imaging. For erlotinib, the PBPK model predicted the TBR compared to the observed ratio less accurately: only 16.6% of TBR was predicted within 3-fold of the observed values. The lung was predicted accurately with a PE of −58.8%. However, the predicted TBR of spleen, kidney, bone and tumor were underestimated (Table 2).

### 2.3. Sensitivity Analysis

The influence of including only a pH-driven approach for lysosomal sequestration (excluding lysosomal membrane-binding distribution [26] (Supplemental Materials S-III, Equation (S7)) in the PBPK model was simulated. The correct tumor-to-lung contrast was not simulated by using this simplified approach for lysosomal sequestration, indicated by the increase in PE for both afatinib and osimertinib (Afatinib PE 45% to 93%, Osimertinib: −6.3% to +27%; see Supplemental Materials S-IV, Table S1). Although the PE decreased for lung TBR in afatinib, tumor TBR remained relatively similar, leading to a worse outcome when tumor-to-lung contrast was simulated.

The influence of EGFR on the mechanistic PBPK model was researched by simulation of the PBPK model without EGFR binding. This PBPK model without EGFR was not able to capture the right tumor-to-lung contrast for mainly afatinib (PE 44% to −62%). Contrary to the observed contrast, without EGFR target binding a higher uptake in lung than in tumor was predicted for afatinib. (Supplemental Materials S-IV, Table S1) The PBPK model including EGFR was able to capture the image quality by predicting the right predictive values in 33.3% of tissues for afatinib, 100% (osimertinib) and 16.6% (erlotinib), within 3-fold of the observed values (Supplemental Materials S-IV, Table S1).

Immune deprivation in the tumor tissue may lead to less macrophages and type II cells in the tumor core, influencing distribution to the tumor. In the final model, this is corrected by adding the fraction F_cell_ to healthy lung tissue, but not to tumor tissue, simulating immune deprivation in tumor tissue. To analyze whether this difference in immune cell presence plays a role in determining drug distribution, we added the same fraction of immune cells to the tumor tissue as well. This model showed a worse outcome when compared to the mechanistic model as described above (Supplemental Materials S-IV), leading to a decrease in accuracy predicting afatinib tumor tissue (PE 124% to 130%) and a decrease in accuracy predicting tumor-to-lung contrast for both afatinib (PE 45% to 53%)) and osimertinib (PE −6.3% to 24%, Supplemental Materials S-IV, Table S1). Since erlotinib is a weak base, immune deprivation was not simulated.

Using the mechanistic PBPK model, we hypothesized that not just perfusion but also vascularization of the tumor determines tumor drug penetration. Histological analysis of the healthy lung tissue samples and adenocarcinoma samples yielded a vasculature coefficient of 0.36, indicating that tumor tissue shows approximately 2.8 times less vessels per mm^2^ tissue than lung tissue. We assumed that all three EGFR TKIs were perfusion independent [15,20]. The influence of the variability in vascularization between tumor and nontumorous lung tissue was tested by including this vasculature reflection coefficient. The prediction of lung uptake decreased by including this parameter for all TKI, compared to the final model, presuming unaltered tumor perfusion (Supplemental Materials S-IV, Table S1, PE −90 to −152%, 45% to −56% and −6.3 to −99% for erlotinib, afatinib and osimertinib, respectively). Therefore, only the perfusion coefficient and not the vasculature coefficient was retained in the final mechanistic PBPK model.

The extracellular water of the tumor microenvironment might become more acidic, leading to a pH shift from 7.4 to 6.7. To determine the influence of this pH change, a simulation was used. Therefore, the model was simulated including the pH change and without. The results showed that taking into account the more acidic extracellular water resulted in more precise prediction. Excluding the more acidic tumor microenvironment mainly affected erlotinib (PE −90 to −93%). The effect on afatinib and osimertinib was minimal (PE 44 to 44% and PE −6.3 to −7.5%, respectively).

## 3. Discussion

The PBPK model in this study was developed to capture essential features of tissue distribution by extending previously published physicochemical base models with EGFR binding and lysosomal sequestration and tumor immune deprivation at unrestricted tumor perfusion [8,9,19]. The developed mechanistical PBPK model was able to capture the right whole-body distribution with a high tissue distribution for osimertinib and afatinib and low tissue distribution and high whole blood concentrations for erlotinib. Furthermore, the model captured the right tumor-to-lung contrast for all EGFR-TKIs and therefore was able to predict image quality.

^11^C-erlotinib reached relatively high concentrations in the blood compared to tissues (TBR < 1), compared to ^18^F-afatinib and ^11^C-osimertinib. This relates to a small whole-body volume of drug distribution, which is similar to the volume of distribution estimated at therapeutic dose levels (erlotinib 232 L, afatinib 2370 L and osimertinib 918 L) [12,27,28]. We have shown that our PBPK model based on physicochemical drug properties of the TKIs predicted these differences in distribution profiles. Furthermore, the negative tumor-to-lung contrast as seen by ^11^C-osimertinib is also predicted by the PBPK model including these parameters.

For osimertinib, we hypothesized that a lower lysosomal volume in tumors, assuming an immune suppressive microenvironment, would lead to a decreased cellular concentration of osimertinib compared to lung tissue. Indeed, this resulted in a correctly predicted tumor-to-lung contrast for osimertinib. Since the decrease in lysosomal sequestration mainly impacts the tumor uptake (Table 2), we showed that the low tumor-to-lung contrast for osimertinib may be explained by immune deprivation and subsequent decrease in lysosomal volume in tumor tissue. The same phenomenon was observed for other TKIs such as nintedanib where increased lysosomal number and lysosomal size decreases sensitivity toward these drugs [29]. This hypothesis is further strengthened by the sensitivity analysis where immune deprivation is excluded from the model. This model was less accurate in predicting uptake, indicating that immune cells play a significant role in tissue uptake.

For afatinib, the predicted decrease in lysosomal sequestration in tumor compared to lung was accompanied by a relative high percentage of EGFR binding (Table 2). For all three compounds, the tumor-to-lung contrast was predicted adequately after accounting for EGFR binding in the model. For afatinib, EGFR binding had the highest influence on tumor distribution in the mechanistical PBPK model due to its low dissociation constant (K_D_) [9,10]: EGFR binding showed the highest contribution to the overall tissue uptake. As shown in the sensitivity analyses, when EGFR binding is removed, tumor-to-lung contrast was highly underpredicted. However, tumor-tracer uptake of erlotinib and osimertinib was underpredicted by our model and for most tissues, erlotinib predictions fell outside of the 3-fold range. We hypothesize that the variation in EGFR abundance and target affinity among patients’ tumors relates to results in high variability in tumor-tracer uptake. Erlotinib and osimertinib EGFR binding may be underpredicted as affinity for wild-type EGFR was applied. Previous research from our group provided the framework for EGFR binding in tissue by demonstrating the ability of PET/CT to distinguish between wild-type and mutated EGFR [17,18,20]. Therefore, future studies should include EGFR-binding affinities for mutated and wild-type receptors, specifically for drugs with differences in affinity between wild-type and mutation.

The distribution of drugs into tissues with high drug transporter abundancy, e.g., brain, kidney and spleen, was less accurate. For erlotinib, only lung was predicted correctly. The underpredictions of the other tissues of interest are best explained by the effect of influx transporters. Erlotinib is a substrate for the influx transporters OAT3 and OCT2 [23]. The influence of the influx transporter OAT on tissue distribution of erlotinib was investigated in a ^11^C-erlotinib PET imaging study in rats. The OAT influx transporter was inhibited by rifampicin and decreasing erlotinib exposure was measured in the kidneys and liver, but the exposure in lung was unaffected [30]. In contrast, the overprediction of the TBR for the brain of afatinib may be caused by drug efflux by MDR1 and the BCRP [31]. These drug efflux transporters are highly abundant in the blood–brain barrier (BBB). Similar to the observations in our PET study, a preclinical permeability study showed a low brain-to-blood ratio of 0.31 for afatinib [31]. Further studies of brain uptake can be performed after extension of the PBPK model to include the central nervous system (CNS) physiologically and brain tumor compartments, as described by Hirasawa et al. [32]. In future studies CNS physiology and both influx and efflux transport processes should be studied and research into how to optimally implement these processes is needed.

Another possible explanation for underprediction of erlotinib is that albumin binding may not be the only process affecting tissue distribution of weak bases to be accounted for. Multiple lipophilic-basic drugs bind with a high affinity to the immune-activated protein alpha-1-acid glycoprotein (AAG) [33]. Prior studies show that in NSCLC patients’ plasma, AAG levels are increased, but little is known of AAG in the extracellular water of tissues in cancer. In lysosomal-rich tissues such as lung, AAG levels may potentially be higher compared to the immune-suppressive microenvironment of the tumor, leading to differences in tissue distribution. As the role of AAG in plasma binding and drug transport of weak bases has been established, further research on the role of AAG for tissue distribution is needed [34]. Finally, the binding of TKIs to the lysosomal membrane had an important influence on the TBR (Assmus vs. Schmitt). The degree of binding to the membrane was based on the compound logP. However, Pearce et al. (2018) showed in their research that the usage of the actual membrane affinity (instead of logP) is more accurate to predict the KPU [35]. They also presented a critical analysis on how to predict a membrane affinity, in case it is not available.

Sensitivity analysis demonstrates the need for inclusion of the lysosomal membrane, since the correct tumor-to-lung contrast was not captured for all compounds when only a pH-driven approach was included in the PBPK model (Schmitt vs. Assmus, [26,36]). The high impact of lysosomal sequestration (Table 1) after microdosed PET may be due to the unsaturated lysosomes. Fluoxetine, a basic lipophilic compound with comparable physicochemical properties (log P = 3.2, pKa = 9.8) to afatinib and osimertinib, shows that at prolonged exposure of therapeutic doses, lysosomal saturation curve occurs [37]. When extrapolating the results to therapeutic PK, potential saturation of lysosomes needs to be accounted for. Additionally, further research into nonlinear processes of drug binding and sequestration may improve the model predictions [24,38], when comparing microdose and therapeutic dose PK.

In the sensitivity analysis, vascularization-driven drug penetration was studied as a hallmark of NSCLC tumors [39,40]. Our results show that vascularization does not drive drug penetration since the model performs worse when this component was added. Previous results have shown that tracer uptake is perfusion-independent. As our research shows that vasculature is reduced in NSCLC, this may result in hypoxia in some regions with reduced O_2_ perfusion within cancers and a decreased capacity to deliver nutrients or remove metabolic waste from rapidly proliferating cells and increased utilization of glucose and the production of lactate [41]. Prior studies show that while the intracellular water pH is highly balanced, extracellular pH in the cells in the tumor microenvironment decreases, resulting in increased uptake of strong basic compounds [41]. The sensitivity analysis showed that the more acidic extracellular water in tumor tissue has only a minor influence on the tumor drug uptake. However, adding this change resulted in a more precise prediction for all three TKIs.

In this study, we extended previously published physicochemical base models with EGFR binding, lysosomal sequestration and more acidic extracellular tumor water. These base models were validated with in vivo data obtained at steady state therapeutic drug concentrations. The effect of each component was studied using sensitivity analyses, but uncertainty of the parameter was not included. Uncertainty evaluation could help predict variability in drug penetration between individuals and is a topic for further research. The PET imaging data used in this study was obtained 1–2 h after a single microdose. Therefore, it should be noted that discrepancies between predicted and observed TBR may occur due to lower drug exposure and nonsteady state. Another study limitation is that drug metabolism and elimination were not taken into account. The half-life of the three researched EGFR-TKIs was comparable and greatly exceeded the 1–2 h scan period (erlotinib = 36 h; afatinib = 45 h; osimertinib = 49 h) [12,27,28], making the assumption of absence of elimination reasonable. Prior research of midazolam, a compound with similar metabolic profile to erlotinib, suggests that metabolism at microdose level is not different from metabolism at therapeutic dose level [42]. However, when these models are applied for predicting the whole-body distribution and target uptake of new tracers with shorter half-lives, inclusion of metabolism and elimination might be needed.

To our knowledge, this is the first PBPK study that addresses these environmental differences. Prior studies either did not account for variability in tumor vs. normal tissue or used an in vitro based partitioning coefficient (e.g., 0.73 in colon cancer [43]). However, other hallmarks of cancer such as the collagen matrix and its effect on the penetration of drugs should be studied.

### Future Perspective

If drug properties can be used to predict differences in image quality, it may be possible to predict tracers with an optimal image quality: drugs with large tumor/tissue contrast. Before applying these predictions in drug development, prospective validation of the predictive value of the PBKP model using new tracers is needed. Furthermore, to predict TBR in future studies more precisely, both active and passive influx and efflux transport needs to be included in the mechanistic PBPK model. Therefore, further in vitro research into binding affinities for transporters and transporter tissue concentrations is needed.

In order to study differences in tumor-to-lung contrast and whole-body distribution between microdose and therapeutic dose, the mechanistic PBPK model needs to be extended in a concentration-dependent manner. The injected dose of ^11^C-erlotinib corresponded with 2.2 µg (±0.46) erlotinib. When compared to the regular therapeutic dose of 150 mg, this is a >10,000-fold difference [20]. With this difference in dosing, lysosomal sequestration, albumin, lipoprotein, AP^−^ and EGFR binding and EGFR target binding will become saturated, and by including these nonlinear processes, the influence of different doses on TBR can be assessed.

The impact of mutational status on tumor-to-lung contrast and whole-body distribution can be investigated by the use of affinity constants for EGFR wild-type and mutated EGFR. First, the activating EGFR mutation needs to be identified in order to use the right affinity value, thereby increasing accuracy of the prediction of tumor uptake. When such models are fully validated, and combined with optimized individual imaging-based uptake measurements, they may predict individualized dosing regimens intended to optimize drug exposure at the site of disease, thereby improving drug efficacy.

## 4. Materials and Methods

### 4.1. Overview

**Figure 3:**
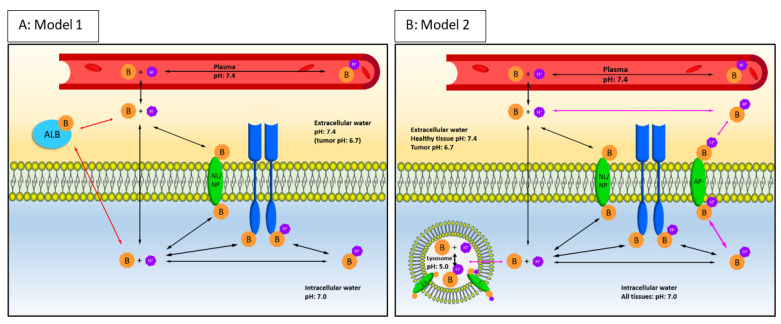
Schematic overview of the final mechanistic PBPK models for weak basic or acidic EGFR-TKIs (**A**: Model 1) and strong basic EGFR-TKIs (**B**: Model 2). The plasma compartment is depicted in red, extracellular space in yellow and intracellular space in blue. Orange hexagons “B” (base) depict the drug; purple hexagons depict H+ atoms. When depicted together, bases are protonated. When depicted separately, the base is unprotonated. Blue receptors depict epidermal growth factor receptor (EGFR), green ovals over the cell membranes are neutral (phospho)lipids (NL/NP) and acidic phospholipids (AP-). Light blue ALB is a representation of albumin. Black arrows depict processes that are included in both models, red/purple arrows depict processes specific for each model. pH values for each compartment are given. Equations for model 1 and 2 below and the model structure are further explained in the Supplemental Materials S-II,III. B = basic unprotonated drug, BH+ = protonated drug, AP- = acidic phospholipids, NL = neutral lipids PL = phospholipids, ALB = albumin, EGFR = epidermal growth factor receptor.

Our goal was to develop a PBPK model that captures essential features of tissue distribution of the three EGFR TKI. First, the base model that best describes the researched drugs was identified based on physicochemical properties. Key components (identified in prior research [21]) were added to the base model: EGFR target binding, lysosomal sequestration of strong bases (osimertinib and afatinib), tumor immune deprivation and unaltered perfusion. Last, we validated our mechanistical PBPK model using data from prior PET research by our group and evaluated whether reduced tumor vascularity could influence drug penetration. All equations and detailed explanation can be found in Supplemental Materials S-I, and an overview of included parameters can be found in Table 3 and schematic overview in Figure 3.

Model 1 full equation:

$$pTBR=\left(F_{vasc/perf}*\left(Kpu\left(1\right)+EGFR\;binding\right)\right)*\frac{Fu}{B:P}$$

Model 2 full equation:

$$pTBR=\left(F_{vasc/perf}*\;\left(Kpu\left(2\right)+EGFR\;binding+\left(\frac{1+10^{pKa-pHiw}}{1+10^{pKa-pHp}}\right)*Kpu,lys*F_{lys}*F_{cell\;type}\right)\right)*\frac{Fu}{B:P}$$

### 4.2. Scan Data

All PET scans were performed in advanced stage, EGFR mutated NSCLC patients. No patients were treated with the treatment analog of the PET tracer prior to scanning (e.g., a patient undergoing ^11^C-erlotinib scanning was treatment-naïve for erlotinib). The PET data used in this research are derived from static, 40–60 min post-tracer dose whole-body PET/CT scans. All regions of interest were delineated by the same experienced researcher in a standardized manner using in-house-developed software. For all tracers, spleen, kidney, tumor, lung (contralateral from tumor site) and vertebra were included. For afatinib and osimertinib, brain was additionally delineated, but erlotinib scans did not include brain tissue in field of view. The full-scan protocol and evaluation of the biodistribution is currently under submission.

### 4.3. PBPK Model: Base Model Selection

The biodistribution of basic lipophilic drugs such as the three researched EGFR-TKIs has been described extensively by well-established PBPK models [45,46]. The PBPK models from Rodgers et al. provide the most accurate tissue distribution predictions [45,46,47]. Choice of model is dependent on compound properties, most importantly basicity. Relevant physicochemical properties used in PBPK modeling of the three EGFR TKIs, erlotinib, afatinib and osimertinib, can be found in Table 3. Since erlotinib is a weak base and osimertinib and afatinib are strong basic drugs, two base models are used. Model 1 is applicable for predicting tissue uptake of the weak basic drugs and was used for erlotinib [45]. Model 2 can be used for afatinib and osimertinib predictions since this model applies to strong basic compounds [46].

The following assumptions were made in all models, as validated by Rogers et al.: drug transport into tissues only occurs passively; conditions are nonsaturating; the drug is at steady state and well-stirred in all tissues of interest; metabolism and drug clearance are negligible at the time of scanning (at <0.05 of the biological half-life); the tissue PET scans did not contain a significant number of blood vessels (in the PBPK model, only the concentration outside of the blood perfusing the tissue was calculated).

In the PBPK model, we focused on tissues in the PET field of view that are large enough to allow adequate PET data analysis (regions of interest > 1.5 cm). The liver was excluded since all EGFR TKI included in this study are metabolized in the liver, leading to unreliable uptake assessments.

### 4.4. Base Models: Physicochemical Drug Distribution

In the physicochemical model, tissue-to-blood ratios (predicted TBR, pTBR) are predicted based on distribution to albumin (ALB), neutral lipids and phospholipids (NL/NP), acidic phospholipids (AP-) and to cellular spaces such as the extra- and intracellular water (EW/IW). The described physicochemical base models predict pTBR at steady state by inclusion of drug-specific physicochemical properties and tissue composition (Table 3). If available, drug-specific properties were adapted from the PET imaging data, such as the blood-to-plasma ratio. Physicochemical properties including pKa values were retrieved from the same in vitro research publication to prevent insecurities and enable comparison of the outcomes [31], and can be found in Table 3. All formulas used in the base models and subsequent additions can be found in Supplemental Materials S-II,III.

Base model 1, the model reflecting weak bases, predicts the pTBR by calculation of the pH-driven distribution to cellular components (Figure 3B). Tissue-specific fractional tissue volumes of cellular components, including intracellular water, extracellular water, neutral lipids and neutral phospholipids are reflected by, respectively, *F_iw_, F_ew_, F_nl_* and *F_np_*. By use of the pH values of the cellular components intracellular water, neutral lipids and neutral phospholipids *pH_iw_, pH_nl_, pH_np_* relative to the pH of plasma (*pH_p_)*, the fraction of unprotonated drug available for diffusion to these cellular parts is predicted. The pH values of the cellular components are shown in Figure 3. The octanol/water partition coefficient (P) is included for binding affinity of the unprotonated drug to neutral lipids and phospholipids in the cell membrane. Since a weak base such as erlotinib is highly (99%) unprotonated in plasma, albumin binding in the extracellular water is a predominant process of tissue distribution. The albumin binding was predicted based on the multiplication of the association constant (Ka) for albumin (Figure 1D) with the tissue specific albumin tissue-to-blood ratio [46]. The formula for calculation of Ka and the base model equations can be found in Supplemental Materials S-II (base model 1, Equations (S1)–(S4)). A schematic overview of base model 1 is depicted in Figure 3A.

In model 2, the model reflecting strong bases, the pTBR contains the same elements for the distribution to neutral (phospho) lipids, intracellular and extracellular water. In contrast to weak bases, afatinib and osimertinib are strong basic drugs (pKa > 7) and are mostly protonated (respectively, 98% and 86%) at physiological pH levels [31], as shown in Figure 3A. This protonation leads to binding to acidic phospholipids (AP-) (Figure 1C). Distribution to acidic phospholipids was predicted using association constant Ka, Supplemental Materials S-II Equation (S4) and tissue-specific concentration [AP-]. Model 2 equations can be found in Supplemental Materials S-III (base model 2, Equations (S5)–(S10)). A schematic overview of model 2 is depicted in Figure 3B.

### 4.5. Extension of the Physicochemical Base Models with EGFR Target Binding

Only nonspecific binding is described by the physicochemical base models. Intracellularly, TKIs bind with high affinity to EGFR [6,9,44]. Differences in affinity of EGFR-TKIs for their target may influence tissue binding and is therefore an essential feature for tissue distribution of TKIs. By adding EGFR binding to base models 1 and 2, target binding was included in the PBPK model. Tissue-specific EGFR concentrations ([EGFR]) and drug-specific dissociation constants (Kd) for wild-type EGFR are shown in Table 3. For two tissues of interest, bone and brain, which lacked relevant literature data, we assumed EGFR was not present.

### 4.6. Extension of the Physicochemical-EGFR Models with Lysosomal Sequestration (Mechanistical PBPK Model)

Because of the protonated status in an environment with physiological pH, the lysosomal trapping was added to the physicochemical base model for strong bases (model 2) only [26,36]. To estimate the binding, the same composition was assumed for the lysosomal membrane as for the outer membrane of the cell. Since immune cells, mostly consist of a higher lysosomal volume and a lower lysosomal pH than normal tissue cells, tissue-specific cell types were included to predict the TBR [48].

### 4.7. Including Hallmarks of NSCLC

Four of the hallmarks of NSCLC tumors are a potential immune-suppressive microenvironment and erratic (and potential inadequate) neovascularization and perfusion caused by changes in the microenvironment [39,40,49] resulting in decrease in pH of the tumor microenvironment [41]. We hypothesized that either of these hallmarks could predict a decreased cellular concentration of the TKIs, even at a high affinity and higher expression of EGFR in the tumor.

The impact of the acidic tumor environment was added. Based on prior research, the pH extracellular water in tumor cells was set at 6.7, while intracellular pH was not altered compared to surrounding lung tissue (Table 3, Supplemental Materials S-III Equation (S12)) [41].

The impact of the lysosomal volume of different cell types on tissue uptake in tumor compared to normal lung was researched. Lung-tissue uptake was simulated by use of a physiological composition including the different immune cells: 4.1% alveolar macrophages, 8.3% type II cells and 87.6% residual cells (Table 3) [36]. To reflect an immune-suppressive microenvironment, tumor-tissue uptake with input parameters concerning only residual lung cells was applied (Table 3, Supplemental Materials S-III Equations (S6) and (S10)).

As a final step in the modeling, we hypothesized that the number of vessels drives drug penetration. The vascular coefficient was calculated by dividing the microvessel density (MVD) of normal lung tissue (4 samples), obtained from the Human Protein Atlas, by the MVD of 8 samples of adenocarcinoma NSCLC patients. MVD was calculated per surface area of CD31+ vessels and tissues. A full description of this analysis can be found in Supplemental Materials S-V. Since tumor uptake of ^11^C-erlotinib and ^18^F-afatinib has previously been shown to be independent of tumor perfusion, we assumed that all three EGFR TKIs were perfusion independent (50).

### 4.8. Simulation of Tumor-to-Lung Contrast and Tissue Distribution

For all EGFR-TKIs, the tumor-to-lung contrast was estimated by dividing the uptake in tumor by the uptake in lung (contrast = pTBR tumor/pTBR lung). This contrast was subsequently validated with the PET imaging data. Furthermore, tissue distribution was assessed by predicting the TBR of the lung, tumor, spleen, kidney, brain and bone, and compared to PET imaging tissue uptake data. The functions of the systemic level are as described in the Supplemental Materials S-II,III.

### 4.9. Software and Statistics

R software (version 4.0.3; R Foundation for Statistical Computing, Vienna, Austria) was used for simulations of the PBPK models and graphical visualization of the predictions and PET observations. PET-TBR data were used to validate the developed PBPK models [21].

The accuracy of mechanistic PBPK model predicted tumor-to-lung contrast and the TBR was assessed by determination of the percentage of tissues falling within 3-fold of the observed data, as is done in the referenced research by Rodger et al. [45,46]. This was researched by calculating prediction errors (PE) and mean prediction error of the mechanistical PBPK model and of subsequent sensitivity analyses. The strength of correlation between the predicted and the PET image-observed TBR was assessed by the Pearson correlation coefficient and with a two-sample *t*-test significance of the correlation:

$$PE=\left(\frac{PRED-OBS}{mean\;\left(PRED+OBS\right)}\right)*100\%\;\;Pearson\;R=\frac{1}{n-1}\sum \;(\frac{x-\bar{x}}{s})\;\left(\frac{y-\bar{y}}{s}\right)$$

Sensitivity analyses were performed by researching the impact of extension with EGFR binding, use of a different lysosomal extension models on tissue-to-blood ratios and the effect of tumor immune deprivation on all tissues of interest. The effect of the aberrant tumor vasculature was determined by comparing the results after inclusion of a vascular versus the (unaltered) perfusion coefficient. When in sensitivity analyses the removal of the extension of the base model showed a significant decrease in predictivity (the mean PE decrease of more than 10% in tumor-to-lung contrast of all three models), the component was retained in the final mechanistic PBPK model.

## 5. Conclusions

Our mechanistic PBPK model consisting of a base model—EGFR binding, lysosomal sequestration, tumor immune deprivation, a changed tumor microenvironment, unaltered tumor perfusion and dependent on physicochemical properties of the relevant drug—was able to accurately predict tumor-to-lung contrast. We therefore conclude that our mechanistical PBPK model accurately predicts image quality for EGFR expressing NSCLC tumors, while further study of distribution for drugs into tissues with high drug transporter abundancy and the effect of EGFR mutation on drug penetration is needed.

## Figures and Tables

**Table 1:** Contribution of the different components in the final mechanistic PBPK models to the predicted TBR in the lung and the tumor.

	Erlotinib	Afatinib	Osimertinib
**Lung**			
EGFR binding	0.21%	16.73%	0.14%
Lysosomal trapping	n.a.	49.19%	59.16%
NL/NP	1.12%	0.01%	0.00%
Albumin	73.01%	n.a.	n.a.
AP-	n.a.	32.63%	39.74%
IW	14.54%	1.08%	0.72%
EW	0.36%	0.36%	0.23%
**Tumor**			
EGFR binding	1.89%	72.17%	1.85%
Lysosomal trapping	n.a.	11.99%	42.79%
NL/NP	1.05%	0.00%	0.00%
Albumin	72.32%	n.a.	n.a.
AP-	n.a.	14.63%	52.91%
IW	13.71%	0.48%	0.96%
EW	11.02%	0.73%	1.49%

NL/NP neutral (phospho)lipids AP- acidic phospholipids IW intracellular water EW extracellular water.

**Table 2:** PET-image-derived tissue-to-blood ratios compared to predicted TBR in all tissues of interest. SD is given in brackets.

	Erlotinib			Afatinib			Osimertinib		
	Predicted	Observed	Prediction Error (%)	Predicted	Observed	Prediction Error (%)	Predicted	Observed	Prediction Error (%)
Brain	0.13	n.a.	n.a.	2.85	0.08 (0.03)	189.7	1.52	0.79 (0.5)	62.7
Lung	0.28	0.51 (0.2)	−58.8	6.89	2.54 (1.2)	92.4	3.11	7.01 (1.6)	−77.1
Spleen	0.17	1.46 (0.4)	−157.8	48.72	13.23 (2.3)	114.6	25.33	18.09 (7.7)	33.3
Kidney	0.21	1.69 (0.6)	−155.6	26.20	6.93 (1.8)	116.3	10.48	5.61 (2.0)	60.6
Bone	0.14	1.23 (0.2)	−158.3	2.72	4.81 (2.0)	−55.3	1.48	4.24 (0.7)	−96.6
Tumor	0.30	1.42 (0.5)	−131.1	15.36	3.60 (2.4)	124.1	2.33	5.60 (2.0)	−82.4

**Table 3:** Tissue- and compound-specific input parameters. Tissue-specific parameters were adapted from Table 1 in Rodgers et al., 2005, Table 1 in Rodgers et al., 2006 and Table 1 in Schmitt et al., 2021, EGFR concentrations from Table 3 in Glassman et al., 2016, and lung-specific parameters from Table 1 in Assmuss et al., 2017.

Tissue-Specific Input Parameters																				
		F_nl_		F_np_			F_ew_		F_iw_			F_lys_^2^		Tissue Concentration of AP- (mg/g) ^2^				Albumin Tissue to Plasma Ratio ^3^		EGFR (nM)
Blood cells		1.7 × 10^−3^		0.0029			n.a.		0.60			n.a.		0.50				n.a.		n.a.
Bone		0.017		0.0017			0.1		0.35			n.d.		0.67				0.10		n.a.
Brain		0.039		0.0015			0.16		0.61			0.014		0.40				0.048		n.a.
Kidney		0.039 ^1^		0.012 ^1^			0.27		0.47			0.017		2.44 ^1^				0.13		177
Lung ^3^		0.0088 ^1^		0.0030 ^1^			0.34		0.43			0.015		0.57 ^1^				0.21		31.1
Tumor												0.01								299
Spleen		0.021 ^1^		0.017 ^1^			0.21		0.53			0.053		3.18				0.097		54.6
Plasma ^4^		n.a.		n.a.			n.a.		n.a.			n.a.		n.a.				n.a.		n.a.
**Lung-specific parameters ^2^**																				
			**F_nl_**		**F_np_**			**F_ew_**			**F_iw_**		**pH_ew_**			**F_lys_**	**pH lysosome**		**F_cell type_**	
-Alveolar macrophages			0.0088^1^		0.0030^1^			0.34			0.45		7.4			0.078	4.75		0.041	
-Type II cells																0.03	5.1		0.083	
-Residual cells																0.01	5.1		0.88	
**Tumor-specific input parameters**																				
Residual cells			0.008		0.0030			0.34			0.45		6.7			0.01	5.1		1	
**Compound-specific parameters**																				
	**Erlotinib**					**Afatinib**				**Osimertinib**					**References**					
Log P	3.3					3.6				3.2					Colclough et al. (2021) [31]					
pKa	5.5					8.2				9.0					Colclough et al. (2021) [31]					
B:P ratio ^5^	0.95					1.27				0.79					Van de Stadt et al. (2021) [21]					
Kd EGFR (nM)	2164					2				155					Joly-Tonetti et al. (2021) [44]					
F_unbound_^6^	0.088					0.095				0.017					Colclough et al. (2021) [31]					

^1^ Translation factor from rats to human [45,46]. ^2^ Input parameter only used in model 1. ^3^ Input parameter only used in model 2; Lung pHew: 7.22; pHp 7.4; pHiw 7.0; pHlys: 5.3. ^4^ Hematocrit (H): 0.45. ^5^ Blood-to-plasma concentration ratio. ^6^ Unprotonated fraction [31]. F_iw_, F_ew_, F_nl_ and F_np_ reflect tissue-specific fractional tissue volumes of the cellular components intracellular water, extracellular water, neutral lipids and neutral phospholipids. Flys, pHlys and Fcell reflect lysosomal volume fraction, lysosomal pH and the fraction of various cell types in tissue. Fvasc: 0.36, Supplemental Materials S-V, and Fperf: 1 reflect the vascular and perfusion coefficient in the tumor compared to the surrounding lung tissue.

## Data Availability

Data is contained within the article and supplementary material.

## Supplementary Materials

The following are available online at https://www.mdpi.com/article/10.3390/ph15070796/s1, S-I: Physiologic equations used to describe the physicochemical parameters, S-II: Components of the mechanistical PBPK-model for weak bases (model 1), S-III: Components of the mechanistical PBPK-model for strong bases (model 2), S-IV: Sensitivity analyses, S-V: Extension Rodgers’ base model, S-VI: PET scan data.
