# Supplementary material for: Physiologically Based Pharmacokinetic (PBPK) Modeling to Predict PET Image Quality of Three Generations EGFR TKI in Advanced-Stage NSCLC Patients
Source: Pharmaceuticals (Basel). 2022 Jun 27;15(7):796. doi: 10.3390/ph15070796 (PMC9315544; doi:10.3390/ph15070796)
Supplement: Supplementary file 1 [file pharmaceuticals-15-00796-s001.zip › Manuscript_PBPK_Pharmaceuticals.V2_supl.pdf]

Physiologically-based pharmacokinetic (PBPK) modeling to predict PET image quality of three generations EGFR TKI in advanced-stage NSCLC patients

Supplements

I.H. Bartelink[1*], E.A. van de Stadt[2*], A.F. Leeuwerik[1], V.L.J.L. Thijssen[3], J.R.I. Hupsel[1], J.F. van den Nieuwendijk[1], I. Bahce[2], M. Yaqub[4], N.H. Hendrikse[4]

1: Department of Clinical Pharmacology and Pharmacy, Amsterdam UMC location VUmc, Amsterdam, the Netherlands

2: Department of Pulmonology, Amsterdam UMC location VUmc, Amsterdam, the Netherlands

3: Department of Radiation Oncology, Amsterdam UMC location AMC, Amsterdam, the Netherlands

4: Department of Radiology and Nuclear Medicine, Amsterdam UMC location VUmc, Amsterdam, the Netherlands

* Contributed equally

Running title: PBPK modeling of EGFR TKI in NSCLC

I. Bartelink

Department of Clinical Pharmacology and Pharmacy

Amsterdam UMC, location VUmc

De Boelelaan 1117

1081HV Amsterdam, the Netherlands

+3120-4444444

i.bartelink@amsterdamumc.nl

Supplemental A

I: Physiologic equations used to describe the physicochemical parameters

a) $$Fraction\ BH^{+} = \frac{1}{1+10^{pKa-pH}}$$

b) $$pTBR_{pHiw}\ or\ contribution\ pH = \left(\frac{1+10^{pKa-pHiw}}{1+10^{pKa-pHp}} * fiw\right) * \frac{Fu}{B:P}$$

c) $$pTBR_{AP-}\ or\ AP\text{-}\ binding = \left(\frac{Ka*[AP-]*10^{pKa-pHiw}}{1+10^{pKa-pHp}}\right) * \frac{Fu}{B:P}$$

d) $$pTBR_{ALB}\ or\ Albumin\ binding = \left(Ka, ALB * \frac{[ALBUMIN]tissue}{[ALBUMIN]p}\right) * \frac{Fu}{B:P}$$

e) $$pTBR_{Lipids}\ or\ Lipid\ binding = \left(\frac{P*Fnl+(0.3P+0.7)*Fnp}{1+10^{pKa-pH}}\right) * \frac{Fu}{B:P}$$

f) $$pTBR_{EGFR}\ or\ EGFR\ binding = \left(\frac{\frac{[EGFR]}{Kd}*(1+10^{pKa-pHiw})}{1+10^{pKa-pHp}} * fiw\right) * \frac{Fu}{B:P}$$

II: Components of the mechanistical PBPK-model for weak bases (model 1)

1. $$Kpu(1) = \left[\left(\frac{1+10^{pKa-pHiw}}{1+10^{pKa-pHp}} * fiw\right) + few + \left(\frac{P*Fnl,t+(0.3P+0.7)*Fnp,t}{1+10^{pKa-pHp}}\right) + (Ka, albumin * [ALBUMIN], tissue)\right]$$

2. $$EGFR\ binding = \left(\frac{\frac{[EGFR]}{Kd}*(1+10^{pKa-pHiw})}{1+10^{pKa-pHp}} * fiw\right)$$

3. $$Ka, albumine = \left[\left(\frac{1}{Funbound} - 1 - \left(\frac{P*Fnl,p+(0.3P+0.7)*Fnp,p}{1+10^{pKa-pHp}}\right)\right) * \left(\frac{1}{[ALBUMIN]p}\right)\right]$$

Full whole-body PBPK equation:

4. $$pTBR = \left(F_{vasc/perf} * (Kpu(1) + EGFR\ binding)\right) * \frac{Fu}{B:P}$$

1: F_{iw}, F_{ew}, $F_{ew,tumor}$, F_{nl}, and F_{np} reflect tissue-specific fractional tissue volumes of the cellular components intracellular water, extracellular water, extracellular water tumor, neutral lipids and neutral phospholipids. By use of the pH values of these cellular components pH_{iw}, pH_{ew}, $pH_{ew,tumor}$, pH_{nl}, pH_{np} relative to the pH_p of plasma, the fraction unprotonated drug available for diffusion to these cellular parts is predicted. pH values of the cellular components are shown in **Figure 2.** The

octanol/water partition coefficient (P) is included for binding affinity to neutral lipids and phospholipids. A 30% lipophilic and 70% hydrophilic ratio was assumed for phospholipids and therefore P is weighted with 0.3. There is no pH partitioning included for the extracellular water since the pH does not differ from the plasma pH. Except for tumor tissue, where $pH_{ew,tumor}$ is used. Albumin binding is a predominant process of tissue distribution since weak bases are highly unprotonated in plasma and will hardly interact electrostatically with the negative loaded acidic phospholipids. Therefore, AP binding is not included in the equation. The albumin binding is predicted based on the multiplication of the association constant (Ka) for albumin with the tissue specific albumin tissue-to-plasma ratio.

2: Tissue-specific EGFR concentrations ([EGFR]) and drug-specific dissociation constants (Kd) for EGFR are included. The intracellular binding of the drug (protonated or unprotonated) was calculated as function of the amount of unbound drug in intracellular water, corrected by the fractional tissue volume, and multiplied by the [EGFR] and inverse Kd

3: The Ka value for albumin included in equation 1 was estimated by equation 3. The estimation was based on the concentration of albumin in plasma ([ALBUMIN]p) multiplied by the inverse of the $F_{unbound}$ subtracted by the partitioning to neutral lipids and phospholipids. The partitioning to neutral lipids and phospholipids was predicted in the same way as for equation 1. The terms $F_{nl,p}$ *and* $F_{np,p}$ refers to fractional volumes of plasma.

4: pTBR is predicted tissue-to-blood ratio. F_{vasc} is the vascular coefficient derived as shown in appendix V. F_{perf} is the perfusion coefficient. F_u is the fraction unbound drug and B:P blood to plasma partition coefficient.

III: Components of the mechanistical PBPK-model for strong bases (model 2)

5. $$Kpu(2) = \left[\left(\frac{1+10^{pKa-pHiw}}{1+10^{pKa-pHp}} * fiw\right) + few + \left(\frac{Ka*[AP-]*10^{pKa-pHiw}}{1+10^{pKa-pHp}}\right) + \left(\frac{P*Fnl,t+(0.3P+0.7)*Fnp,t}{1+10^{pKa-pHp}}\right)\right]$$

6. $$EGFR\ binding = \left(\frac{\frac{[EGFR]}{Kd}*(1+10^{pKa-pHiw})}{1+10^{pKa-pHp}} * fiw\right)$$

7. $$Kpu,lys = \left[\left(\frac{1+10^{pKa-pHlys}*fiw}{1+10^{pKa-pHiw}} * fiw\right) + \left(\frac{Ka*[AP-]*10^{pKa-pHlys}}{1+10^{pKa-pHiw}}\right) + \left(\frac{P*Fnl+(0.3P+0.7)*Fnp}{1+10^{pKa-pHp}}\right)\right]$$

8. $$Ka,AP = Kpu,bc - \left(\frac{1+10^{pKa-pHbc}}{1+10^{pKa-pHp}} * fiw,b\right) - \left(\frac{P*Fnl,b+(0.3P+0.7)*Fnp,b}{1+10^{pKa-pHp}}\right) * \left(\frac{1+10^{pKa-pHp}}{[AP-]*10^{pKa-pHbc}}\right)$$

9. $$Kpu,bc = \left(\frac{H-1+(B:P)}{Funbound*H}\right)$$

Full whole-body PBPK equation:

10. $$pTBR = \left(F_{vasc/perf} * \left(Kpu(2) + EGFR\ binding + \left(\frac{1+10^{pKa-pHiw}}{1+10^{pKa-pHp}}\right) * Kpu,lys * F_{lys} * F_{cell\ type}\right)\right) * \frac{Fu}{B:P}$$

5: F_{iw}, F_{ew}, $F_{ew,tumor}$, F_{nl}, and F_{np} reflect tissue-specific fractional tissue volumes of the cellular components intracellular water, extracellular water, neutral lipids and neutral phospholipids. By use of the pH values of these cellular components pH_{iw}, pH_{ew}, $pH_{ew,tumor}$, pH_{nl}, pH_{np} relative to the pH_p of plasma, the fraction unprotonated drug available for diffusion to these cellular parts is predicted. pH values of the cellular components are shown in **Figure 1.** The octanol/water partition coefficient (P) is included for binding affinity to neutral lipids and phospholipids. A 30% lipophilic and 70% hydrophilic ratio was assumed for phospholipids and therefore P is weighted with 0.3. There is no pH partitioning included for the extracellular water since the pH does not differ from the plasma pH. Except for tumor tissue, where $pH_{ew,tumor}$ is used. Since afatinib and osimertinib are both predominantly protonated at physiological pH levels, albumin binding is not included in this model.

6: Tissue-specific EGFR concentrations ([EGFR]) and drug-specific dissociation constants (Kd) for EGFR are included.

7: The partitioning to the lysosomal membrane include binding to acidic phospholipids, neutral lipids and phospholipids and was predicted in the same way as for equation 5. The same composition was assumed for the lysosomal membrane as for the outer membrane of the cell. Tissue specific cell types were included to predict the tissue-to-plasma ratio (Eq. 10). F_{lys} and $F_{cell,type}$ reflect the fractional lysosomal volume and the fraction of the specific cell type. Immune cells, like the macrophage, mostly consist of a higher lysosomal volume and a lower lysosomal pH than normal tissue cells.

8: The partitioning into red blood cells (Kpu,bc) was predicted by use of the hematocrit (H), the blood-to-plasma (B:P) ratio and the fraction unbound in plasma $F_{unbound}$ (Eq. 9). Subsequently, in equation 8 the Ka for AP was calculated by subtract partitioning to other cellular components from the Kpu,bc. The partitioning to cellular components was calculated in the same way as for equation 5. However, for equation 8 the terms $F_{iw,b}$, $F_{nl,b}$ *and* $F_{nl,b}$ refers to fractional volumes of the red blood cell. Since red blood cells do not contain lysosomes, inclusion of lysosomal partitioning was not needed. Assumed was that the found Ka for AP in red blood cells reflects the Ka for AP for all tissues throughout the body

9: pTBR is the predicted tumor to blood ratio. Fcell type reflect the fraction of the various cell types in lung and tumor vary in the extent of lysosomal sequestration (Flys) In tumor we simulated only (100%) residual cells and in lung the following fractions: 4.1% alveolar macrophages, 8.3% type II cells and 87.6% residual cells. Fvasc is the tumor vasculature reflection coefficient to reflect the reduced difference in tissue drug penetration by neovascularization in the tumor compared to the surrounding lung tissue (**Supplement V**), and Fperf is the tumor perfusion coefficient which reflect unaltered tissue drug penetration by the micro-environment of the tumor compared to the surrounding lung tissue

IV: Sensitivity analyses

Supplementary table 1: Sensitivity analyses of extensions of the final mechanistic PBPK-model . The model includes physicochemical drug distribution, lysosomal sequestration, tumor immune deprivation and unaltered tumor perfusion and EGFR target binding, but excludes vascularization. 1) Final model with only pH of the lysosome, but without membrane lysosome (Schmitt et al vs Asmuss et al(1, 2)) 2) final model without EGFR binding 3) final model without tumor immune deprivation 4) final model with tumor vascularization & 5) final model without pH adjustment for tumor extracellular water. Predicted TBR, observed PET TBR and the predicted vs observed Tumor-to-lung ratio are shown.

TL-ratio: Tumor-to-lung ratio

			Erlotinib			Afatinib			Osimertinib		
			Lung	Tumor	TL-ratio	Lung	Tumor	TL-ratio	Lung	Tumor	TL-ratio
Final	Mechanistic PBPK-model	Predicted	0.28	0.30	1.06	6.89	15.36	2.23	3.11	2.33	0.75
		Observed	0.51	1.42	2.78	2.54	3.60	1.42	7.01	5.60	0.80
		PE (%)	**-58.77**	**-131.11**	**-89.59**	**92.37**	**124.06**	**44.41**	**-77.12**	**-82.33**	**-6.26**
Sensitivity analysis	1) model - membrane lysosome	Predicted				3.50	13.52	3.86	1.27	1.33	1.05
		PE (%)				**31.95**	**115.90**	**92.51**	**-138.67**	**-123.02**	**27.29**
	2) model – EGFR	Predicted	0.28	0.29	1.04	5.74	4.28	0.75	3.10	2.29	0.74

	**PE (%)**	**-58.97**	**-132.19**	**-90.95**	**77.37**	**17.17**	**-62.27**	**-77.24**	**-83.93**	**-7.98**
**3) model – tumor immune deprivation**	**Predicted**				6.89	16.91	2.45	3.11	3.17	1.02
	**PE (%)**				**92.37**	**129.79**	**53.44**	**-77.12**	**-55.36**	**24.35**
**4) model – addition vascularization**	**Predicted**	0.28	0.106	0.38	6.89	5.53	0.80	3.11	0.84	0.27
	**PE (%)**	**-58.77**	**-172.13**	**-151.73**	**92.37**	**42.29**	**-55.50**	**-77.12**	**-147.84**	**-98.92**
**5) Tumor – addition acidic pH extra cellular water**	**Predicted**		0.28	1.02		15.28	2.22		2.31	0.74
	**PE (%)**		**-133.38**	**-92.79**		**165.97**	**44.41**		**47.51**	**-7.45**

V: Extension Rodgers' base model

We hypnotized that Rodgers' base model to calculate the TBR was not accurate enough. Based on literature and the hall marks of Cancer a few extensions of the model were done and analysed. As mentioned in the sensitivity analysis. The extensions which are discussed in this paper are: Histological analysis of vasculature, intra cellular EGFR binding and pH swift of tumor extra cellular water. The background to our decision making is discussed below.

Va: histological analysis of vasculature

CD31 staining is widely used to quantify neovascularization since CD31 is abundantly found on the surface of endothelial cells. Quantitative evaluation of vascularization was performed by analysis of images (obtained from the Human Protein Atlas (www.proteinatlas.org) of immunohistochemical CD31 staining of lung tissue. Microvessel density (MVD) was then determined by counting the number of vessels per tissue area. To obtain the vascularization coefficient, ratio of MVD was divided by the mean MVD of healthy tissue. The analysis included eight adenocarcinoma and four normal lung tissue samples. Mean MVD of tumor tissue was 85 (± 36) and for healthy tissue mean MVD was 237 (±74). The vasculature coefficient of NSCLC was therefore 85/237 = 0.36. **Supplemental figure 1** shows the MVD of all analyzed samples.

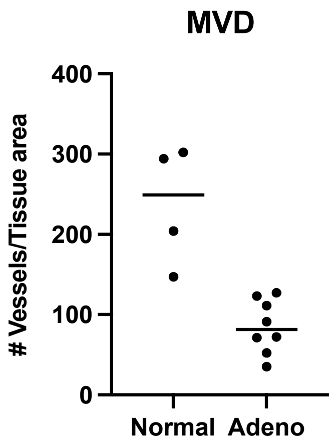

Supplemental figure 1: MVD of each sample. # =number of. Normal = healthy tissue. Adeno = adenocarcinoma, tumor tissue. MVD= micro vessel density

Vb: Contribution EGFR

The contribution from EGFR has been taken into account following Rodgers' way of adding the parameters. Only what is present in the intracellular water (IW) can bind to EGFR. We assumed that B and BH^+ equally bind to EGFR. So, the total unbound concentration in the intracellular water ($\boldsymbol{C,iw}$) can bind to EGFR, according to Rodgers that equals:

$$(1 + 10^{pKa-pHiw}) * fiw$$

The amount that actually binds to EGFR is dependent on the concentration ($[\mathbf{EGFR}]$) and the affinity for EGFR ($\boldsymbol{Ka = \left(\frac{1}{Kd}\right)}$). Addition to the $\boldsymbol{C,iw}$ results in the concentration bound to EGFR ($\boldsymbol{C}_{\text{EGFR}}$):

$$C_{\text{EGFR}} = \left(\frac{[EGFR]}{Kd}\right) * \left(1 + 10^{pKa-pHiw}\right) * fiw$$

The tumor to plasma water partition coefficient ($\mathbf{Kpu}$) is calculated as follows:

$$Kpu = \left(\frac{C{,}tissue}{C{,}p}\right)$$

The concentration in plasma $\boldsymbol{C,p}$ is defined as:

$$1 + 10^{pKa-pHp}$$

Substituting $\boldsymbol{C}_{\text{EGFR}}$ and $\boldsymbol{C,p}$ into the equation for the $\boldsymbol{Kpu}$ results in the contribution of the binding to EGFR, to the total $\boldsymbol{Kpu}$:

$$\text{Kpu}_{\text{EGFR}} = \frac{\left(\frac{[EGFR]}{Kd}\right) * (1+10^{pKa-pHiw}) * fiw}{1+10^{pKa-pHp}} = \frac{\left(\frac{[EGFR]}{Kd}\right) * (1+10^{pKa-pHiw})}{1+10^{pKa-pHp}} * fiw$$

After addition to the model of the weak bases the final equation becomes:

$$\boldsymbol{Kpu(2)} = [\left(\frac{(1 + 10^{pKa-pHiw}) * fiw}{1 + 10^{pKa-pHp}}\right) + few + \left(\frac{P * Fnl,t + (0.3P + 0.7) * Fnp,t}{1 + 10^{pKa-pHp}}\right)$$

$$+ \ (Ka, albumine \ * \ [Albumine, tissue])$$

$$+ \left(\frac{\left(\frac{[EGFR]}{Kd}\right) * (1 + 10^{pKa-pHiw}) * fiw}{1 + 10^{pKa-pHp}}\right)$$

Do mind that in this elucidation the parts that cancel each other out in the final equation have been left out. Rodgers et al. assumed that at steady-state [B,iw] = [B,p], therefore in actuality C,iw and C,p are not defined as : $\mathbf{C, iw = (1 + 10^{pKa-pHiw}) * fiw}$ and $\mathbf{C, p = (1 + 10^{pKa-pHp})}$ but as $\mathbf{C, iw = ([B, iw] * (1 + 10^{pKa-pHiw})) * fiw}$ and $\mathbf{C, p = ([(B, p] * (1 + 10^{pKa-pHiw})) * fiw}$.

VC: pH extra cellular water tumor

KPU tumor

Tumor tissue differs from healthy tissue, one of these differences is the pH of the extra cellular water. In healthy tissue the extra cellular water has a pH of 7.4, the same as blood plasma. However, the tumor tissue has a more acidic pH of 6.7. As a result, drugs undergo a different protonation in tumor tissue compared to healthy tissue. The base model of Rodgers' (2005&2006) takes only healthy tissue into account. The main assumption done by Rodgers' is that at steady state, the unbound un-protonated drug is equal on both sides of a membrane. Because of this, the concentration unbound and un-protonated drug in plasma equal the concentration in extra cellular water (eq. 1). Consequently, Cu_P can be used as the concentration which is able to diffuse into the intra cellular space, instead of the concentration $C_{U,EW}$. This yields Rodgers' final equation, from which the intra cellular part is shown in equation 2.

$$Cu_P = C_{U,EW} * \left(\frac{\cancel{1 + 10^{pKa-pH_{EW}}}}{\cancel{1 + 10^{pKa-pH_P}}}\right) \qquad (1)$$

$$C_{U,IW} = Cu_P * \left(\frac{1 + 10^{pKa-pH_{IW}}}{1 + 10^{pKa-pH_P}}\right) \qquad (2)$$

In the case of tumor tissue and the more acidic pH of extra cellular water, Cu_P is no longer equal to $C_{U,EW,tumor}$. This results in a new equation for the concentration unbound drug in extra cellular water (eq. 3)

$$C_{U,EW,tumor} = Cu_P * \left(\frac{1 + 10^{pKa-pH_{EW}}}{1 + 10^{pKa-pH_P}}\right) \qquad (3)$$

The extra cellulair space is yielding a higher drug concentration. Although, there is still a steady-state, besides there is an equilibrium. As a result, $Cu_p = Cu_{iw,tumor} = Cu_{ew,tumor}$. When $Cu_{iw,tumor} = Cu_{ew,tumor}$ is used instead of $Cu_p = Cu_{iw,tumor}$, follows equtation (4).

$$C_{U,IW,tumor} = Cu_{EW,tumor} * \left(\frac{1 + 10^{pKa-pH_{IW}}}{1 + 10^{pKa-pH_{EWt}}}\right) \qquad (4)$$

Equation 3 can be substituted in equation 4, which yields equation 5, the new equation for the concentration unbound drug in intra cellular water of the tumor.

$$C_{U,IW,tumor} = \left(Cu_P * \left(\frac{\cancel{1 + 10^{pKa-pH_{EWt}}}}{1 + 10^{pKa-pH_P}}\right)\right) * \left(\frac{1 + 10^{pKa-pH_{IW}}}{\cancel{1 + 10^{pKa-pH_{EWt}}}}\right) \ (5) \rightarrow C_{U,IW,tumor} = Cu_P * \left(\frac{1 + 10^{pKa-pH_{IW}}}{1 + 10^{pKa-pH_P}}\right)$$

As showed above, the change in pH of extra cellular water does not change the concentration unbound and un-protonated drug in the intra cellular water. Therefore, the concentration bound to neutral lipids, neutral phospholipids and acidic phospholipids is also not affected by the pH change. On the contrary, drugs are only able to bind to albumin in the extra cellular water. An increase in drug in the extra cellular spaces means more available drug to bind to albumin. this is shown in equation 6.

$$Contribution\ albumin = \left(\frac{1}{fu} - 1 - \frac{\left(P * f_{NL,P} + \left((0.3P + 0.7) * f_{NP,P}\right)\right)}{1 + 10^{pKa-pH_{plasma}}} * \frac{[ALB]_T}{[ALB]_P} * \left(\frac{1 + 10^{pKa-pH_{EWt}}}{1 + 10^{pKa-pH_P}}\right)\right) \ (6)$$

All changes together yield the following equations for tumor tissues:

$$KpuR_{Tumor(1)} = \frac{C_T}{Cu_P} = \left(\left(\frac{1 + 10^{pKa-pH_{EWt}}}{1 + 10^{pKa-pH_P}}\right) * f_{EW} + \left(\frac{1 + 10^{pKa-pH_{IW}}}{1 + 10^{pKa-pH_p}} * f_{IW}\right) + \left(\frac{1}{fu} - 1 - \frac{\left(P * f_{NL,P} + \left((0.3P + 0.7) * f_{NP,P}\right)\right)}{1 + 10^{pKa-pH_{plasma}}} * \frac{[ALB]_T}{[ALB]_P} * \left(\frac{1 + 10^{pKa-pH_{EWt}}}{1 + 10^{pKa-pH_P}}\right)\right) + \left(\frac{P * f_{NL} + \left((0.3P + 0.7) * f_{NP}\right)}{1 + 10^{pKa-pH_p}}\right)\right) \ (7)$$

$$KpuR_{Tumor\,(2)} = \frac{C_T}{Cu_P} = \left(\left(\frac{1 + 10^{pKa-pH_{EWt}}}{1 + 10^{pKa-pH_P}}\right) * f_{EW} + \left(\frac{1 + 10^{pKa-pH_{IW}}}{1 + 10^{pKa-pH_p}} * f_{IW}\right) + \left(\frac{K_a * [AP^-] * 10^{pKa-pH_{IW}}}{1 + 10^{pKa-pH_p}}\right) + \left(\frac{P * f_{NL} + \left((0.3P + 0.7) * f_{NP}\right)}{1 + 10^{pKa-pH_p}}\right)\right) \ (8)$$

Due to the fact that TKI's bind to EGFR, we added the contribution of EGFR binding on the KPU and TBR to Rodgers' base model using equation 9.

$$Contribution\ EGFR\text{:}\ \frac{\left(\frac{[EGFR]}{Kd}\right) * \left(1 + 10^{pKa-pHiw}\right)}{1 + 10^{pKa-pHp}} * fiw \quad (9)$$

TKI's can only bind to EGFR in the intra cellular water, the concentration drug in intra cellular tumor water is not affected by the pH swift, so the contribution EGFR is also not affected

Following Assmus, we added lysosomal trapping to the KPU eqation for strong bases. Only unbound and un-protonated drug can diffuse from the intra cellular water into the lysosomes, which was not effected by the extra cellular water pH change.

$$\boldsymbol{Contribution\ lysosome_{tumor}} = \sum_{cell\ type\ 1}^{cell\ type\ n} \left(\frac{1 + 10^{pKa-pH_{EWt}}}{1 + 10^{pKa-pHp}} \right) * \boldsymbol{KPU_{lys}} * \boldsymbol{f_{lys}} * \boldsymbol{f_{cell\ type}} \quad (10)$$

Where KPU_{lys} is given by equation 11. Note that no alternations are done to equation 11, due to the fact that KPU_{lys} is based on pH differences between intra cellular water and the lysosomes.

$$\boldsymbol{KPU_{lys}} = \left(\frac{1+10^{pKa-pH_{lysosome}}}{1+10^{pKa-pH_{iw}}} * \boldsymbol{f_{iw}} + \frac{K_a*[AP^-]_T*10^{pKa-pH_{lysosome}}}{1+10^{pKa-pH_{iw}}} + \frac{\left(P*f_{NL}+((0.3P+0.7)*f_{NP})\right)}{1+10^{pKa-pH_{iw}}} \right) \quad (11)$$

All taken into account yield the final KPU_{tumor} equation (12): This is the addition of equation (6)/(7) with equation (9) and (10):

$$\boldsymbol{KPU_{tumor(1/2)}} = \left(\boldsymbol{KpuR_{tumor(1/2)}} + \left(\frac{\left(\frac{[EGFR]}{Kd}\right) * \left(1 + 10^{pKa-pHiw}\right)}{1 + 10^{pKa-pHp}} * \boldsymbol{fiw} \right) + \sum_{cell\ type\ 1}^{cell\ type\ n} \left(\frac{1 + 10^{pKa-pH_{EWt}}}{1 + 10^{pKa-pHp}} \right) * \boldsymbol{KPU_{lys}} * \boldsymbol{f_{lys}} * \boldsymbol{f_{cell\ type}} \right) * \frac{\boldsymbol{Fu}}{\boldsymbol{B:P}} \quad (12)$$

VI: PET scan data

	Erlotinib	Afatinib	Osimertinib
Number of patients	8	7	4
Average injected dose (Mbq)	387 ± 23	350 ± 34	323± 79
Included tumors	12	12	6
Age (years, sd)	69.8 (0.46)	63.28 (11.7)	64.8 (17.8)
Gender (% Female)	46	43	50
Scan type	Static	Static	Static

1. Schmitt MV, Lienau P, Fricker G, Reichel A. Quantitation of Lysosomal Trapping of Basic Lipophilic Compounds Using In Vitro Assays and In Silico Predictions Based on the Determination of the Full pH Profile of the Endo-/Lysosomal System in Rat Hepatocytes. Drug Metab Dispos. 2019;47(1):49-57.

2. Assmus F, Houston JB, Galetin A. Incorporation of lysosomal sequestration in the mechanistic model for prediction of tissue distribution of basic drugs. Eur J Pharm Sci. 2017;109:419-30.
